# Gender Disparity in the Risk of Hypertension in Subjects With Major Depressive Disorder

**DOI:** 10.3389/fpsyt.2019.00541

**Published:** 2019-08-02

**Authors:** Wei-Tsung Kao, Chen-Lin Chang, Chi-Hung Lin, Shang-Liang Wu, Shang-Lun Lin, For-Wey Lung

**Affiliations:** ^1^Laboratory of Research, Kaohsiung Armed Forces General Hospital, Kaohsiung, Taiwan; ^2^Department of Psychiatry, Kaohsiung Armed Forces General Hospital, Kaohsiung, Taiwan; ^3^Graduate Institute of Medical Science, National Defense Medical Center, Taipei, Taiwan; ^4^Department of Nursing, Shu Zen Junior College of Management and Medicine, Kaohsiung, Taiwan; ^5^Chi-hung Clinic, Kaohsiung, Taiwan; ^6^School of Medicine, Griffith University, Gold Coast, Australia; ^7^Calo Psychiatric Center, Pingtung County, Taiwan

**Keywords:** major depressive disorder (MDD), propensity score matching (PSM), hypertension, risk factors, National Health Insurance Database

## Abstract

**Objects:** The aim of our study was to investigate whether major depressive disorder (MDD) increased the risk of hypertension using propensity score matching (PSM) in patients with MDD in Taiwan.

**Methods:** In this study, we recruited all samples from a random sample sub-dataset of one million insured individuals from 2005. A total of 743,114 outpatients were included in our study. We used PSM (nearest neighbor matching) stratified by age, hospital level, insurance amount, and Charlson Comorbidity Index score.

**Results:** The hazard ratio (HR) of hypertension was significantly greater in the male MDD outpatients (HR = 1.116, *P* = 0.004) than in the female MDD outpatients (HR = 0.93, *P* = 0.02). Using PSM, we selected 27,988 outpatients with hypertension and 27,988 outpatients without hypertension for a nested case–control study. In this analysis, female outpatients with MDD (relative risk = 0.852) had lower risks of hypertension. Male outpatients without/with MDD (relative risk = 1.987/3.018) showed a synergistic interaction with gender in which male patients had a higher risk of hypertension in a multiplicative model. Furthermore, MDD appeared to have an interaction effect with gender (HR = 1.82, *P* < 0.001) in the proportional hazards model analysis. Antidepressant use also increased the risk of hypertension (HR = 1.16, *P* < 0.001).

**Conclusions:** There was gender disparity in the risk of hypertension in subjects with MDD. MDD outpatients who used antidepressants had a higher risk of suffering from hypertension. A large-scale, population-based study is warranted to generalize these results in the future.

## Background

The World Mental Health Survey estimates that depression affects 350 million people worldwide (1) with a lifetime risk of 7% (2). Depression will become the second global disease burden after ischemic heart disease by 2020. Several studies have shown that major depressive disorder (MDD) is one of the most common mental disorders worldwide, with a prevalence ranging between 2.1 and 7.6% (3–6). In Taiwan, the prevalence of MDD increased from 0.167 to 1.724% from 1996 to 2003, which was lower than the prevalence reported in Western countries (7–10).

Hypertension is associated with at least 7.6 million deaths annually worldwide, which accounts for 13.5% of all deaths (11), and people with hypertension have a higher risk of all types of cardiovascular diseases (12–15). A cross-sectional study (16) of 153,996 adults aged from 35 to 70 years from 17 countries showed that 40.8% of the subjects had hypertension. Another study (17) showed that the prevalence of hypertension in six European countries, Canada, and the USA ranged from 29.8 to 60.2% in men and from 23.8 to 50.3% in women. The prevalence of hypertension in Taiwan was 23.2% (26.5% in men and 19.0% in women) from 1993 to 1996 and decreased to 17.6% (20.9% in men and 14.4% in women) from 2005 to 2008. From 2013 to 2014, the prevalence rate increased to 25.6% (29.6% in men and 21.8% in women) (18).

MDD and hypertension are both important issues in Taiwan and globally. Past studies have shown an association between MDD and hypertension (19–27), although other studies have found no such association (28, 29). Two studies determined that the risk of hypertension was increased years after the occurrence of MDD (24–27).

To the best of our knowledge, only one large population-based study has investigated this topic; this study was a random sample of 1 million individuals registered in the Taiwanese National Health Insurance Database (25). The results are in agreement with those of the present study, which observed an association between MDD (on a 1-year basis) and a subsequent diagnosis of hypertension with an odds ratio (OR) of 1.22. Another study (30) showed that the age-adjusted OR for depression in persons with hypertension was 1.293 (95% CI 1.256–1.331) in the general population using administrative healthcare data from Stockholm County, Sweden. Our previous study (31) showed that hypertension was a possible vulnerability marker for depression in patients with end-stage renal disease.

Based on the above studies, we hypothesize that an association exists between depression and hypertension. The aim of our study was to investigate whether MDD increased the risk of hypertension and to analyze the related risk factors of hypertension among patients with MDD in Taiwan’s National Health Insurance Database.

## Methods

### Data Resources

The National Health Insurance (NHI) system, which is the source of the study data, provides insurance coverage for more than 98% of the 23 million Taiwanese people, and has contracted with more than 93% of medical institutions since 1996. The National Health Research Institute (NHRI) administers all medical claims information recorded from the contracted health care facilities. We retrieved all sampled subjects from the Longitudinal Health Insurance Database (LHID). The LHID includes all original medical claims and registration files for the one million enrollees in the NHI program. The one million random sample enrollees in the LHID were taken from all insured persons registered in the 2015 registry of beneficiaries (*N* = 23.72 million) (32). This dataset contained the registry of medical facilities, orders for inpatients and outpatients, dental services, and prescriptions linked to anonymous identifiers. Thousands of published SCI articles have demonstrated the high validity of NHI data (33–35). Because all patient identifiers were released to the public for research purposes, the LHID was omitted from full review by the Institutional Review Board in Taiwan. However, we obtained an ethics certificate (protocol number 105-049) from the Institutional Review Board of Kaohsiung Armed Forces General Hospital in Taiwan.

### Study Samples

**Figure 1:**
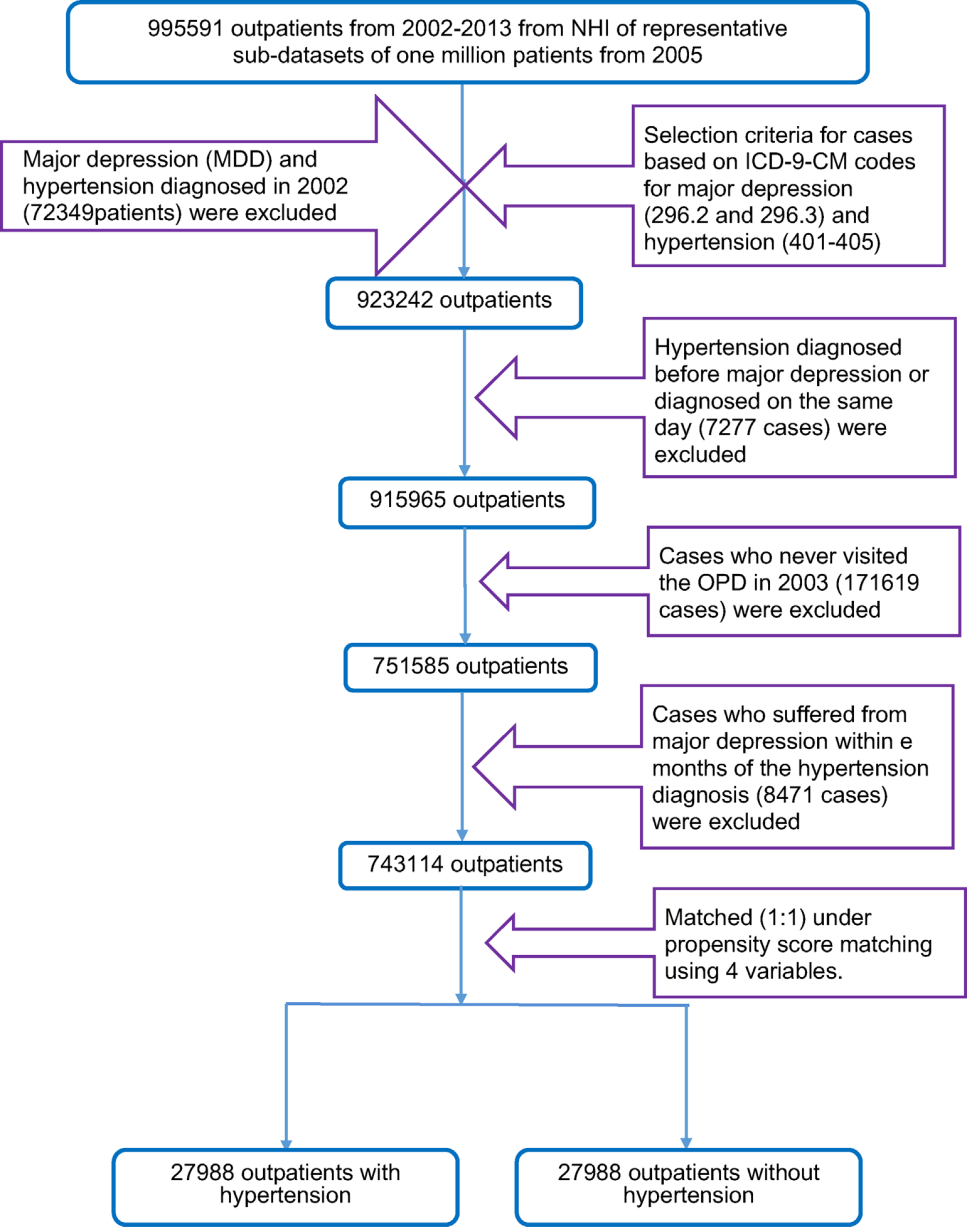
Flowchart of sample selection. Retrospective design for major depression and hypertension.

When selecting samples for analysis in this retrospective study, we first recruited 995,591 outpatients between 2002 and 2013 from a representative one million sub-datasets. We selected the samples using the ICD-9-CM (International Classification of Diseases, 9th revision, Clinical Modification) codes for hypertension (ICD-9-CM codes 401 X-405 X) and major depression (ICD-9-CM codes 296.2X and 296.3X). Our goal was to include new cases in our study, but we could not clarify whether these cases (major depression and hypertension diagnosed in 2002) had visited the outpatient department (OPD) before 2002. Therefore, major depression and hypertension cases diagnosed in 2002 (72,349 patients) were excluded. To explore whether MDD increased the risk of hypertension, MDD needed to occur before hypertension. Therefore, cases in which MDD was diagnosed after hypertension or diagnosed on the same day (7,277 cases) were excluded (**Figure 1**). Because the observations in our study occurred starting in 2003, cases that did not visit the OPD in 2003 (171,619 cases) were excluded. To prevent confusion from cases who suffered from major depression and hypertension over a time span that was too small to determine whether these cases suffered from MDD before hypertension, cases who suffered from hypertension within 3 months of the diagnosis of major depression (8,471 cases) were excluded. Finally, 743,114 outpatients were included in our study. Additionally, we used propensity score matching to select 27,988 outpatients with hypertension and 27,988 outpatients without hypertension.

### Measurements

Information on demographic factors was obtained, including age, gender, antipsychotic use (divided into first- and second-generation antipsychotics), antidepressant use, mood stabilizer use, Charlson comorbidity index (CCI) score, hospital level (divided into medical centers, regional hospitals, district hospitals, and local clinics), and insurance amount. The ICD-9-CM version of the CCI was used to measure the comorbid disease status in the study; this version has been a valuable resource for several health services researchers (36–40). The insurance amount (also known as the insured wage) represented the salaried income and was used instead of the socioeconomic status in our study; this measure was divided into the following three categories: <640 US$, 640–1,280 US$, and >1,280 US$.

### Study Starting Point, Endpoint, and Follow-Up Duration

We defined our starting point, endpoint, and follow-up duration as follows. The follow-up period for the cases was between January 1, 2003 and December 31, 2013. For the cases without major depression, the starting point was the first ambulatory care visit (including outpatient departments of hospitals or clinics); for the cases with major depression, the starting point was the time of the first diagnosis of depression. For the cases with hypertension, the endpoint was the time of the first diagnosis of hypertension; for the cases without hypertension, the endpoint was the last ambulatory care visit. The follow-up duration was calculated from the starting point to the endpoint.

### Statistical Analysis

The data were analyzed using the IBM SPSS Statistics for Windows, Version 21.0 (IBM Corp. Released 2012. Armonk, NY: IBM Corp). Baseline characteristics were compared using an independent *t* test or the chi-square test. Taking into account the factor of follow-up duration, Cox’s regression analysis with hazard ratios (HRs) was used to estimate the association of the risk of hypertension with major depression after adjusting for related variables.

The propensity score exact matching method presented by Thoemmes (41) was adapted in this study. The propensity score matching method is a statistical technique that is used to select samples by capturing the relevant differences between any two units into a single (propensity) score that encapsulates multiple characteristics. This method provides a natural weighting scheme that yields unbiased estimates of the impact by interested factors (42). Variables in the stratum that were matched and controlled included age, CCI score, hospital level, and insurance amount.

## Results

In total, 743,114 outpatients were included in our study, of which 12,570 cases had MDD and 730,544 cases did not have MDD. The *t* test analysis (**Table 1**) found significant differences in age (*T* = 2,341.9, *P* < 0.001) and the CCI score (*T* = 1,702.4, *P* < 0.001) between the MDD and non-MDD groups. The MDD group was older than the non-MDD group. The CCI score was higher in the MDD group than in the non-MDD group. The chi-square test (**Table 1**) found significant differences in gender (***χ*** ^2^ = 1,191, *P* < 0.001), the hospital level visited by the cases (***χ*** ^2^ = 17,384, *P* < 0.001), and the insurance amount (***χ*** ^2^ = 13.44, *P* = 0.001) between the MDD and non-MDD groups. A higher ratio of female patients was noted in the MDD group. Fewer MDD outpatients visited clinics relative to the other hospital levels.

**Table 1:** Comparison of demographic data between the major depressive disorder (MDD) and non-MDD groups.

		MDD group (*n* = 12,570)	Non-MDD group (*n* = 730,544)	*t* value	*χ* ^2^	*P* value
Age (years)		37.8 ± 14.2	29.8 ± 18.4	2,341.9		<0.001
Male		4,138(32.9%)	353,836(48.4%)		1,191	<0.001
CCI^a^		1.2 ± 1.6	0.7 ± 1.3	1,702.4		<0.001
Hospital level^b^	1	3,294(26.2%)	52,372(7.2%)		17,384	<0.001
	2	4,275(34.0%)	68,712(9.4%)			
	3	1,450(11.5%)	61,231(8.4%)			
	4	3,551(28.2%)	548,229(75.0%)			
Insurance amount (US$)^c^	<640	8,488(67.5%)	492,689(67.4%)		13.44	0.001
	640–1,280	2,530(20.1%)	140,658(19.3%)			
	>1,280	1,552(13.3%)	97,197(13.3%)			

a CCI, ICD-9-CM version of the Charlson Comorbidity Index.

b Hospital level: 1, medical centers; 2, regional hospitals; 3, district hospitals; 4, local clinics.

c 1 US$ = 30.1 NT$.

We used proportional hazards model analysis to explore the risk of hypertension for the 743,114 outpatients. **Table 2** showed that the hazard ratio of hypertension did not significantly increase in the MDD group (ICD-9-CM codes 296.2X and 296.3X) (HR = 0.95, *P* = 0.134) after adjusting for other confounders. However, the hazard ratio for hypertension was significantly greater for the male MDD outpatients (HR = 1.12, *P* = 0.004) than for the female MDD outpatients (HR = 0.93, *P* ≤ 0.001) after adjusting for the other confounders. Therefore, we observed an interaction between the MDD and gender (HR = 1.14, *P* = 0.009).

**Table 2:** Proportional hazards model analysis of the MDD and non-MDD groups adjusted for related factors of hypertension.

	Total		Female		Male	
	*P* value	HR (95% CI)	*P* value	HR (95% CI)	*P* value	HR (95% CI)
MDD (yes/no)^a^	0.134	0.95(0.90–1.01)	<0.001	0.93(0.88–0.99)	0.004	1.12(1.04–1.20)
Gender (male/female)	<0.001	1.18(1.16–1.19)				
MDD and gender interaction	0.009	1.14(1.03–1.25)				
Age (years)	<0.001	1.06(1.06–1.06)	<0.001	1.07(1.07–1.07)	<0.001	1.05(1.05–1.05)
CCI^b^	<0.001	1.16(1.16–1.17)	<0.001	1.16(1.15–1.16)	<0.001	1.18(1.17–1.18)
Hospital level (1/4)^c^	<0.001	0.93(0.92–0.95)	<0.001	0.93(0.91–0.96)	<0.001	0.95(0.92–0.97)
Hospital level (2/4)	0.536	1.01(0.99–1.02)	0.266	0.99(0.96–1.01)	0.020	1.03(1.00–1.05)
Hospital level (3/4)	<0.001	1.05(1.03–1.07)	0.001	1.05(1.02–1.08)	<0.001	1.06(1.04–1.09)
Insurance amount (2/1)^d^	<0.001	1.19(1.18–1.21)	<0.001	1.15(1.13–1.17)	<0.001	1.24(1.22–1.27)
Insurance amount (3/1)	<0.001	1.23(1.22–1.25)	<0.001	1.09(1.06–1.11)	<0.001	1.35(1.32–1.37)

a MDD, Major depressive disorder.

b CCI, ICD-9-CM version of the Charlson Comorbidity Index.

c Hospital level: 1, medical centers; 2, regional hospitals; 3, district hospitals; 4, local clinics.

d Insurance amount: 1, < 640 US$; 2, 640–1,280 US$; 3, > 1,280 US$; 1 US$ = 30.2 NT$.

Male (HR = 1.18, *P* < 0.001) and older (HR = 1.06, *P* < 0.001) outpatients had a higher risk of suffering from hypertension. Additionally, outpatients with a higher CCI score (HR = 1.16, *P* < 0.001) had a higher risk of suffering from hypertension. Relative to the local clinics, the outpatients who visited medical centers (HR = 0.93, *P* < 0.001) had a lower risk of suffering from hypertension, whereas the outpatients who visited district hospitals (HR = 1.05, *P* < 0.001) had a higher risk of suffering from hypertension. Relative to an insurance amount <640 US$, outpatients with insurance amounts in the range from 640 to 1,280 US$ (HR = 1.19, *P* < 0.001) and >1,280 US$ (HR = 1.23, *P* < 0.001) both had higher risks of suffering from hypertension.

The mean follow-up time was 9.46 ± 2.64 years (after the first OPD in 2003), the minimum follow-up period was 0.3 years, and the maximum follow-up period was 11.0 years for the 743,114 outpatients.

After propensity score matching, which used nearest neighbor matching by age, CCI score, hospital level, and insurance amount, we selected 27,988 outpatients with hypertension and 27,988 outpatients without hypertension. The demographic data between the two groups are shown in **Supplementary Table 1**. We found that there was no difference between age, CCI score, hospital level, and insurance amount after propensity score matching. Because our aim was to confirm the gender disparity in the risk of hypertension, we did not control for gender in the propensity score matching analysis. Relative to female outpatients without MDD, female outpatients with MDD had a lower risk of hypertension (relative risk = 0.852), whereas male outpatients without MDD (relative risk = 1.987) or with MDD (relative risk = 3.018) had higher risks of hypertension in a multiplicative model (**Table 3**).

**Table 3:** Relative risks^a^ of hypertension among three groups (female outpatients with MDD, male outpatients without MDD, and male outpatients with MDD) in comparison with the female outpatients without MDD for 27,988 outpatients with hypertension and 27,988 outpatients without hypertension (nearest neighbor matching by age, CCI, hospital level, and insurance amount) in a multiplicative model.

		Major depressive disorder	
		No (*N* = 55,413)	Yes (*N* = 563)
Gender	Female	1.000 (8,545/26,003)	0.852 (124/443)
	Male	1.987 (19,200/29,410)	3.018 (119/120)

a Risks are expressed relative to a risk of 1.000 for female outpatients without MDD.

In order to consider the factor of follow-up duration, we also used proportional hazard model analysis to explore the risk of hypertension in the nested case–control study (**Table 4**). We observed that the hazard ratio (HR) of hypertension was significantly greater for male MDD outpatients (HR = 2.69, *P* < 0.001) and female MDD outpatients (HR = 1.53, *P* < 0.001). Additionally, MDD appeared to have an interaction effect with gender (HR = 1.82, *P* < 0.001) in the proportional hazards model analysis. On the other hand, the survival curve of suffering hypertension between four groups (female outpatients without MDD, female outpatients with MDD, male outpatients without MDD, and male outpatients with MDD) are shown in **Supplementary Figure 1**. Male outpatients with MDD had highest risk of hypertension and female without MMD had lowest risk of hypertension. First-generation (*P* = 0.466) and second-generation antipsychotic use (*P* = 0.141) had no significant effects on the risk of hypertension in the total cases. However, second-generation antipsychotic use increased the risk of hypertension in the female outpatients (HR = 1.38, *P* = 0.014). Antidepressant use increased the risk of hypertension in the male outpatients (HR = 1.44, *P* = 0.001), female outpatients (HR = 1.20, *P* = 0.003), and total cases (HR = 1.16, *P* < 0.001) (**Table 4**). Mood stabilizer use only increased the risk of hypertension in the female outpatients (HR = 1.31, *P* = 0.016) and total cases (HR = 1.21, *P* = 0.012) and not in the male outpatients (HR = 1.73, *P* = 0.151).

**Table 4:** Proportional hazards model analysis for 27,988 outpatients with hypertension and 27,988 outpatients without hypertension (nearest neighbor matching by age, CCI, hospital level, and insurance amount).

	Total		Female		Male	
	*P* value	HR (95% CI)	*P* value	HR (95% CI)	*P* value	HR (95% CI)
MDD (yes/no)^a^	<0.001	1.56(1.29–1.88)	< 0.001	1.53(1.25–1.88)	<0.001	2.69(2.20–3.29)
Gender (male/female)	<0.001	2.85(2.77–2.92)				
MDD × gender interaction	<0.001	1.82(1.41–2.35)				
Age (years)	<0.001	1.01(1.01–1.01)	0.001	1.00(1.00–1.01)	<0.001	1.02(1.02–1.02)
CCI^b^	0.906	1(0.99–1.02)	<0.001	0.81(0.78–0.83)	<0.001	1.21(1.16–1.25)
Hospital level (1/4)^c^	0.702	1.02(0.94–1.1)	<0.001	0.67(0.57–0.78)	<0.001	1.63(1.36–1.96)
Hospital level (2/4)	0.323	1.03(0.97–1.1)	<0.001	0.58(0.51–0.66)	<0.001	1.67(1.44–1.92)
Hospital level (3/4)	0.955	1(0.94–1.07)	<0.001	0.59(0.51–0.68)	<0.001	1.54(1.33–1.78)
Insurance amount (2/1)^d^	<0.001	0.93(0.91–0.96)	<0.001	0.85(0.80–0.89)	0.384	1.02(0.95–1.09)
Insurance amount (3/1)	<0.001	0.8(0.78–0.83)	<0.001	0.57(0.53–0.61)	<0.001	0.83(0.78–0.89)
1st antipsychotic use^e^	0.466	1.04(0.93–1.16)	0.446	1.07(0.90–1.27)	0.539	1.23(0.87–1.74)
2nd antipsychotic use^e^	0.141	1.13(0.96–1.33)	0.014	1.38(1.07–1.77)	0.992	0.84(0.50–1.44)
Antidepressant use	<0.001	1.16(1.07–1.25)	0.003	1.20(1.07–1.35)	0.001	1.44(1.17–1.76)
Mood stabilizer use	0.012	1.21(1.04–1.4)	0.016	1.31(1.05–1.63)	0.151	1.73(1.01–2.96)

a MDD, major depressive disorder.

b CCI, ICD-9-CM version of the Charlson Comorbidity Index.

c Hospital level: 1, medical centers; 2, regional hospitals; 3, district hospitals; 4, local clinics.

d Insurance amount: 1, < 640 US$; 2, 640–1,280 US$; 3, > 1,280 US$; 1 US$ = 30.2 NT$.

e 1st antipsychotic use: first-generation antipsychotic use; 2nd antipsychotics: second-generation antipsychotic use.

## Discussion

A gender difference in the prevalence of MDD which is more than twice in women than in men has been well-established in multiple studies (43–47). Our study showed a similar result (32.9% in men vs. 67.1% in women) in **Table 1**. We also found that the prevalence of MDD were 1.2% in 357,974 male outpatients and 2.2% in 385,140 female outpatients between 2002 and 2013.

A previous study (25) showed an increased risk of hypertension in patients with major depressive disorder. However, this cross-sectional study only presented a possible association and not a temporal relationship. Although Wu et al. found that the prevalence and incidence of hypertension were higher among MDD patients than among the general population in their case–control study, they could not clarify whether depression occurred before the onset of hypertension or as a consequence of hypertension. On the other hand, they did not consider the factor of follow-up duration. Cox’s regression analysis with hazard ratios (HRs) was not used in their study.

In our study, outpatients who received a diagnosis of hypertension before major depression or diagnoses for both diseases on the same day (7,277 cases) were excluded. Additionally, outpatients who suffered from hypertension within 3 months of the diagnosis of major depression (8,471 cases) were excluded. After these procedures, we confirmed that MDD occurred before the onset of hypertension in our study.

Our study used proportional hazards model analysis to explore risk of hypertension for 743,114 outpatients; the time factor was added to the analysis, and the observation duration was from 2003 to 2013. This approach was superior to the approach used in the previous study (25), which used risk ratios to analyze the prevalence (only in 2005) and incidence (2006–2008) of hypertension in patients with major depression. In the proportional hazards model analysis of the total cases, the results of our study were similar to previous research (48, 49), with male and older subjects having a higher rate of hypertension.

In the retrospective analysis of the male and female outpatients (**Table 2**), we found different effects of MDD on the occurrence of hypertension between the two groups. MDD increased the risk of hypertension in the male outpatients. In contrast, MDD did not increase the risk of hypertension in the female outpatients. **Table 3** shows that female outpatients with MDD had a lower risk (relative risk = 0.852) of hypertension than female outpatients without MDD and that male outpatients with MDD (relative risk = 3.018) had a higher risk than the male outpatients without MDD (relative risk = 1.987). Moreover, the male and female outpatients with MDD both had higher risks of hypertension in the proportional hazards model analysis of the 27,988 outpatients with hypertension and 27,988 outpatients without hypertension after propensity score matching (**Table 4**). In **Table 4**, it indicates that female outpatients with MDD had a higher risk (HR = 1.53, *P* < 0.001) than female outpatients without MDD. The results in **Tables 2** and **3** were the opposite. The possible reasons were discussed below.

However, the use of large samples like in **Table 2** can lead to the following potential issues: 1) studies with large samples are likely to be more sensitive in the detection of significant results, and 2) a large sample size may cause false positives or negatives during the statistical analysis of control charts (50). Hence, the validity of large samples needs to be examined to decrease the type I error (false positives). Therefore, we used propensity score matching (nearest neighbor matching) for four variables in our study to select 27,988 outpatients with hypertension and 27,988 outpatients without hypertension in an effort to prevent issues due to the large sample size. The different effects of MDD on the risk of hypertension among female outpatients before and after propensity score matching (**Tables 2** and **4**) may be a result of this approach.

Even after propensity score matching, different risks of hypertension were observed among female MDD outpatients between the multiplicative model and proportional hazards model analysis (**Tables 3** and **4**), which might be due to control-related variables associated with hypertension and the follow-up duration.

However, the hazard ratio of hypertension was higher in the male MDD outpatients than in the female outpatients, and an interaction was found between MDD and gender in **Table 4**. One possible explanation for this gender disparity among MDD patients and increased hypertension is that sex hormones are responsible for the difference because premenopausal women are relatively protected against hypertension compared to men and postmenopausal women (51). Another possible explanation for the results is gender differences stemming from social factors. Men and women tend to have different perceptions of healthy behaviors, and men tend to have unhealthier lifestyles (i.e., smoking and alcohol consumption) than women. Furthermore, men are less likely to perceive themselves as being at risk for health problems (52–54). In clinical, there were differences in the symptom of depression between men and women (55). Depressive men more commonly had serious symptoms than depressive women. This may also explain that the hazard ratio of hypertension was higher in the male MDD outpatients than in the female outpatients.

Regarding the effects of drug use on the risk of hypertension among patients with MDD, antidepressants (29) and antipsychotics (56) were shown to elevate the risk of hypertension among MDD patients in past studies. One study also showed that antidepressant use increased the risk of hypertension (29). In our study, antidepressant use increased the risk of hypertension in male outpatients, female outpatients, and the total cases. However, the data about different kinds of antidepressants use were not included in our study, so we cannot analyze the effects of different kinds of antidepressants to the risk of hypertension.

## Limitations

Our study had several limitations. Because our cases were selected from the Longitudinal Health Insurance Database (LHID2005), certain risk factors, such as smoking (57, 58) and alcohol consumption (59), could not be included in our analysis. On the other hand, it also led us to select the samples using the ICD-9-CM codes for hypertension and MDD instead of using standardized instrument to diagnose hypertension and MDD. Therefore, a large-scale, population-based study that includes complete risk factors is necessary. Furthermore, large data are likely to be more sensitive when detecting significant results. We used the propensity score matching method to control variables associated with hypertension, and the results before and after propensity score matching were consistent.

## Conclusions

Using proportional hazards model analysis in a retrospective study and a nested case–control study, we observed gender disparity in the risk of hypertension in subjects with MDD. The hazard ratio of hypertension was higher in the male MDD outpatients than in the female outpatients. Furthermore, a large population should be considered in the future to generalize these results.

## Author Contributions

W-TK and C-LC, with help of F-WL, planned the present study’s content and analysis, interpreted the data, and wrote the paper. W-TK, C-LC, C-HL, S-LW, S-LL, and F-WL initiated and performed the whole survey, analyzed the data, and helped to interpret the findings and to write the paper. All authors read and approved the final manuscript.

## Funding

The study was supported by a research grant (넰KAFGH 105-049) from the Outpatient Foundation of Kaohsiung Armed Forces General Hospital (Kaohsiung, Taiwan).

## Conflict of Interest Statement

The authors declare that the research was conducted in the absence of any commercial or financial relationships that could be construed as a potential conflict of interest.
