# Supplementary material for: Gender Disparity in the Risk of Hypertension in Subjects With Major Depressive Disorder
Source: Front Psychiatry. 2019 Aug 2;10:541. doi: 10.3389/fpsyt.2019.00541 (PMC6688710; doi:10.3389/fpsyt.2019.00541)
Supplement: Supplementary file 1 [file Table_1.pdf]

Supplementary Table 1. Comparison of Demographic Data between 27988 Outpatients with Hypertension and 27988 Outpatients without Hypertension (Nearest Neighbor Matching by Age, CCI, Hospital Level and Insurance Amount)

		Outpatients With Hypertension (n= 27988)	Outpatients Without Hypertension (n= 27988)	t-value	χ^2	P-value
MDD		243(0.9%)	320(1.1%)		10.64	0.001
Male		19313(69.0%)	10205(36.5%)		5948	<0.001
Age (yrs)		36.7±11.6	36.7±11.6	0		1.000
CCI*		0.5±0.75	0.5±0.75	0		1.000
Hospital level**	1	674(2.4%)	674(2.4%)		0	1.000
	2	1108(4.0%)	1108(4.0%)			
	3	925(3.3%)	925(3.3%)			
	4	25273(90.3%)	25273(90.3%)			
Insurance amount (US$)***	<640	14881(53.2%)	14881(53.2%)		0	1.000
	640-1280	7390(26.4%)	7390(26.4%)			
	>1280	5709(20.4%)	5709(20.4%)			
1st Antipsychotic use*****		512(1.8%)	439(1.6%)		5.7	0.017
2nd Antipsychotic use*****		213(0.5%)	138(0.8%)		16.13	<0.001
Antidepressant use		1294(4.6%)	1224(4.4%)		2.038	0.153
Mood stabilizer use		270(1.0%)	166(0.6%)		25.00	<0.001

*CCI: ICD-9-CM version of the Charlson Comorbidity Index; **Hospital level: 1: Medical centers, 2: Regional hospitals, 3: District hospitals, 4: Local clinics; ***1 US$=30.1 NT$; *****1st Antipsychotic use: First generation antipsychotic use, 2nd Antipsychotics: Second generation antipsychotic use
