# Supplementary material for: Gender Disparity in the Risk of Hypertension in Subjects With Major Depressive Disorder
Source: Front Psychiatry. 2019 Aug 2;10:541. doi: 10.3389/fpsyt.2019.00541 (PMC6688710; doi:10.3389/fpsyt.2019.00541)
Supplement: Supplementary file 2 [file Image_1.pdf]

Supplementary Figure 1. The survival curve of suffering hypertension between four groups

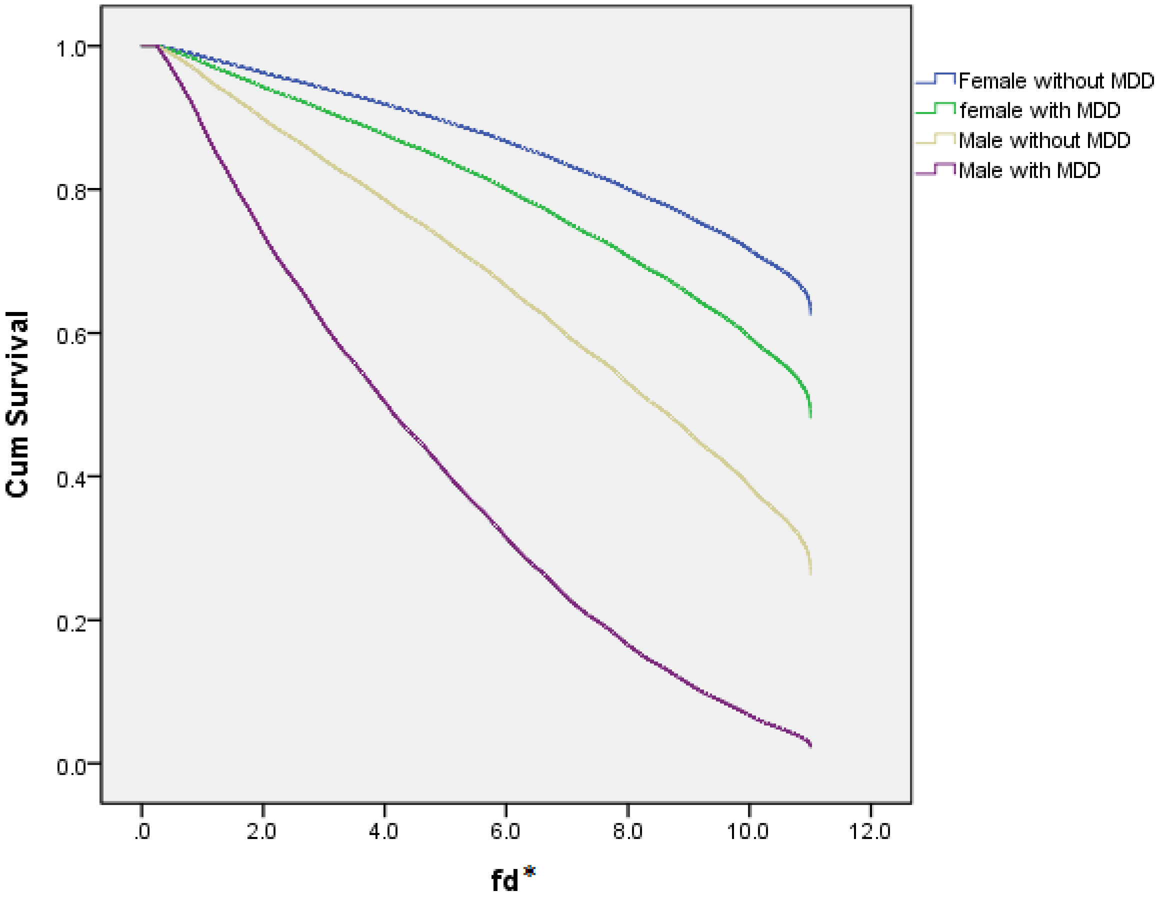

Four groups: Female Outpatients without MDD, Female Outpatients with MDD, Male Outpatients without MDD, and Male Outpatients with MDD; *fd: follow up duration
